# Gene Therapy Techniques and Delivery Methods (Review)

**DOI:** 10.17691/stm2025.17.6.06

**Published:** 2025-12-29

**Authors:** E.I. Shchukina, I.O. Mazunin, I.I. Eremin, A.A. Moskalev

**Affiliations:** Junior Researcher, Institute of Biology of Aging and Healthy Longevity Medicine with Preventive Medicine Clinic; Petrovsky Russian Research Center for Surgery, 2 Abricosovskiy Pereulok, Moscow, 119435, Russia; PhD, Head of the Department of Biology and Genetics, B.V. Petrovsky Medical University; Petrovsky Russian Research Center for Surgery, 2 Abricosovskiy Pereulok, Moscow, 119435, Russia; MD, PhD, Deputy Director for Research; Petrovsky Russian Research Center for Surgery, 2 Abricosovskiy Pereulok, Moscow, 119435, Russia; DSc, Professor, Corresponding Member of the Russian Academy of Sciences, Director of the Institute of Biology of Aging and Healthy Longevity Medicine with Preventive Medicine Clinic; Petrovsky Russian Research Center for Surgery, 2 Abricosovskiy Pereulok, Moscow, 119435, Russia

**Keywords:** gene therapy, CRISPR genome editing, base editing, prime editing, adeno-associated virus vectors, lipid nanoparticles, mitochondrial genome editing, RNA editing, epigenome editing

## Abstract

Gene therapy has evolved into a sophisticated field encompassing diverse precision editing platforms and advanced delivery systems capable of addressing complex genetic disorders and age-related pathologies. This comprehensive review examines the current landscape of gene therapeutic technologies, including CRISPR-based genome editing, base editing systems, prime editing platforms, and emerging DNA polymerase-based editors alongside their corresponding delivery methodologies. The review encompasses viral vectors, including tissue-specific adeno-associated virus serotypes, non-viral delivery systems such as ionizable lipid nanoparticles and virus-like particles, and innovative platforms, including exosome-based delivery and the SEND system. We examine therapeutic applications spanning nuclear genome editing, mitochondrial genome modification, RNA editing, and epigenetic modulation, demonstrating the expanding scope of gene therapy beyond traditional monogenic disorders. Critical analysis reveals that while fundamental technological capabilities have been established, significant challenges remain in manufacturing scalability, long-term safety assessment, delivery across physiological barriers, and optimization of editing efficiency in post-mitotic tissues. The integration of artificial intelligence approaches for predictive analysis and rational vector design represents a promising avenue for addressing current limitations. This review concludes that successful clinical implementation requires systematic resolution of manufacturing, safety, and delivery challenges alongside the development of standardized protocols for patient stratification and robust regulatory frameworks that accommodate rapid technological innovation while ensuring patient safety.

## Introduction

The emergence of precision gene therapy represents a paradigmatic shift in therapeutic intervention, fundamentally altering our capacity to address genetic disorders and age-related pathologies at their molecular origins [1]. Contemporary gene therapy encompasses a sophisticated arsenal of technologies that operate through distinct yet complementary mechanisms: direct genetic correction via programmable nucleases, epigenetic modulation through chromatin-targeted effectors, and functional gene replacement using optimized delivery vectors [2]. The convergence of CRISPR-based genome editing platforms with advanced base editing systems has enabled unprecedented precision in correcting pathogenic mutations, while the development of prime editing and emerging DNA polymerase-based editors has expanded the therapeutic scope to encompass complex genomic rearrangements previously considered intractable [3–5].

As of 2023, 3900 gene therapy clinical trials have been completed in 46 countries [6]. It has reported 7 AAV-based gene therapies approved by the FDA (Luxturna, Zolgensma, Hemgenix, Beqvez, Roctavian, Elevidys, Kebilidi) [7]. The first CRISPR-based therapy Casgevy received regulatory approval in multiple regions for sickle cell disease and beta thalassemia [8].

These technological advances have been paralleled by revolutionary improvements in delivery methodologies, where tissue-specific adeno-associated virus vectors, ionizable lipid nanoparticles, and engineered virus-like particles now provide targeted therapeutic cargo delivery with enhanced safety profiles and reduced immunogenicity [9–11]. Concurrently, the expansion of therapeutic targets to include mitochondrial genomes, RNA transcripts, and epigenetic landscapes has broadened the applicability of gene therapy beyond traditional monogenic disorders to encompass complex age-related conditions characterized by multifactorial pathogenesis [12].

Recent evidence suggests that polygenic traits such as metabolic syndrome and cognitive decline can also be targeted through polygenic genome editing strategies, offering hope for interventions beyond Mendelian disorders [13].

The primary goal of this comprehensive review is to provide a critical analysis of the current state of gene therapy technologies and their delivery systems, examining both their therapeutic potential and the challenges that must be addressed for successful clinical translation. The purpose of this work is to synthesize the diverse technological advances in genome editing platforms with contemporary delivery methodologies, evaluate their clinical applications across different therapeutic contexts, and identify the key barriers that must be overcome to achieve widespread therapeutic implementation.

## Modern strategies of gene therapy and gene editing

Gene therapy approaches can be broadly categorized into two complementary modalities: direct gene delivery and genome editing. The primary gene therapy approach involves delivering functional gene copies or regulatory elements to target cells to restore compromised functions, whereas genome editing enables precise sequence modifications without requiring exogenous gene introduction. In recent years, significant progress has been achieved in developing vectors with enhanced specificity and delivery efficiency, alongside advanced editing platforms including CRISPR/Cas, base editors, and prime editing [14]. These technologies are becoming key tools for precision modification of cellular functions aimed at preventing, slowing, or reversing age-related changes.

Gene therapy relies on the exogenous delivery of functional gene copies to compensate for defective endogenous counterparts. Substantial progress has been made in optimizing expression cassettes and regulatory elements [15, 16]. Tissue-specific promoters and enhancers ensure localized expression, reducing systemic toxicity [17, 18]. Recombinant AAV (rAAV) vectors have been optimized for enhanced transduction efficiency while minimizing insertional mutagenesis risk [19]. Polycistronic constructs with self-cleaving P2A peptides enable co-expression of multiple proteins from a single vector [20].

Current CRISPR-mediated genome editing platforms demonstrate precise gene sequence modifications. Advances in Cas nuclease engineering have produced high-specificity variants (SpCas9-HF1, eSpCas9, HypaCas9) with minimized off-target activity [21]. NTLA-2001 for transthyretin amyloidosis became the first approved *in vivo* CRISPR product, utilizing lipid nanoparticles for delivering ribonucleoprotein complexes with hepatocyte-specific tropism [22]. NTLA-2001 trial in patients with transthyretin amyloidosis with cardiomyopathy demonstrated safety and dose-dependent reduction of serum TTR protein (up to 87%) in patients with hereditary transthyretin amyloidosis after a single intravenous dose [23]. Building on this success, Intellia Therapeutics has advanced NTLA-2002 for hereditary angioedema into Phase III trials, achieving sustained plasma kallikrein reduction of up to 95% with single-dose administration [24].

The 2iHDR (dual inhibition of HDR) technology simultaneously inhibits DNA-dependent protein kinase and DNA Polθ to dramatically improve CRISPR/ Cas9 genome editing precision, achieving up to 80% templated insertion efficiency while minimizing unintended mutations and off-target effects [25].

Additionally, the expanding toolbox of compact genome editors — such as engineered Cas12f and hpCasMINI variants [26, 27] — holds promise for packaging constraints in AAV delivery.

The integration of artificial intelligence with CRISPR technologies is revolutionizing the precision and efficiency of genome editing approaches. AI algorithms are being employed to automate various aspects of CRISPR systems, including guide RNA design optimization, Cas9 variant selection, and prediction of off-target effects. Machine learning models can accurately predict the activity and specificity of different Cas9 variants, allowing for selection of the most suitable enzyme for particular applications while minimizing off-target effects. This computational approach significantly reduces the time and resources required for CRISPR-based research while enhancing safety profiles [28]. Recent advances include the development of OpenCRISPR-1, the first AI-designed CRISPR editor that demonstrates comparable activity to natural systems while being hundreds of mutations away from known proteins [29]. Large-scale screening platforms such as Perturb-seq are being integrated with AI to streamline guide design and functional outcome prediction, bridging the gap between design and phenotypic validation [30]. The TIGER deep learning model now enables precise prediction of both on-target and off-target activity for RNA-targeting CRISPR systems [31].

Base editing fuses a catalytically impaired CRISPR nuclease (most often Cas9 nickase) to a programmable nucleobase-modifying enzyme, allowing single-base conversions (C→T by cytidine deaminases and A→G by engineered adenosine deaminases) in genomic DNA without introducing double-strand breaks, thereby sharply reducing indels and large rearrangements compared with nuclease-based editing. Its toolbox now spans numerous deaminase families — APOBEC1/3, AID, CDA1 for cytosine editing and evolved TadA variants for adenine editing — plus glycosylase-coupled C→G and A→C transversion editors, each engineered with tailored PAM-less Cas proteins, split-inteins, or miniature capsids to widen target scope and fit viral or non-viral delivery constraints [32]. Because productive editing relies only on brief single-stranded DNA exposure within the R-loop and uses nick-directed DNA repair, base editors achieve high on-target efficiencies (often >60%) with dramatically lower off-target activity that can be further minimized through high-fidelity Cas variants, deaminase point mutations, and transient mRNA or RNP delivery. These attributes have facilitated rapid clinical translation, with BE4- and ABE8e-based therapies now undergoing Phase I trials for hemoglobinopathies, cardiovascular disease, and ocular disorders, confirming base editing as a versatile and clinically relevant tool for precise genetic correction and functional modulation [33]. Recent applications have also expanded to immune modulation and *in vivo* liver reprogramming in metabolic diseases, highlighting the systemic therapeutic reach of next-generation base editors [34].

Recently, Cas7-11 systems have been identified as programmable single-component RNA editors with high specificity and low off-target potential in mammalian cells [35]. The first clinical application of CRISPR-Cas13 RNA editing has been approved by the FDA, with HuidaGene’s HG202 therapy for neovascular age-related macular degeneration representing a breakthrough in RNA-targeting therapeutics [36].

The first base editing clinical trial results from Beam Therapeutics’ BEAM-101 for sickle cell disease have demonstrated safety and efficacy, with base editing achieving precise single-nucleotide changes without double-strand breaks [37]. Additionally, Verve Therapeutics has reported successful *in vivo* base editing of PCSK9, achieving up to 59% reduction in LDL cholesterol levels in patients with familial hypercholesterolemia [38].

Prime editing couples a Cas9 nickase to a reverse transcriptase and uses a single prime-editing guide RNA (pegRNA) that both targets the locus and templates the desired change, enabling direct “search-and-replace” of DNA without creating double-strand breaks. This chemistry can install all 12 possible base substitutions as well as small insertions, deletions, and their combinations — capabilities that greatly surpass base editors or nuclease-driven HDR and are restricted mainly by pegRNA design rather than a nearby PAM. Because productive editing demands three sequential RNA-DNA hybridization checkpoints (spacer binding, primer-binding-site annealing, and 3′-flap pairing), prime editors show unusually low off-target activity and produce minimal indels when used in PE2/PE3b formats. A growing toolkit of variants — engineered RTs, PAM-relaxed or mini-Cas domains, split-intein and LNP/ AAV delivery formats, and twin-pegRNA strategies that allow kilobase-scale insertions or deletions — continues to improve efficiency and widens the therapeutic scope of this versatile platform [39].

A recent advancement in prime editing technology, specifically PE4max systems, has shown improved efficiency and reduced unintended DNA changes (indels) by incorporating a strategy that inhibits the DNA mismatch repair system [40]. Prime Medicine has received FDA clearance for PM359, the first prime editing clinical trial targeting chronic granulomatous disease, marking a pivotal milestone in translating this technology to patients [41].

DNA-polymerase editors fuse a nickase-Cas9 to a DNA-dependent polymerase (or tether one in trans) so that the free 3′ end created by the nick primes template-directed synthesis, enabling “search-and-write” editing without double-strand breaks or reverse-transcriptase cassettes. Current formats span (i) error-prone Pol-Cas fusions that saturate mutagenesis windows hundreds of nucleotides long [42], (ii) phage-polymerase systems that copy single-stranded donor templates delivered on a tether [43], and (iii) HUH-nickase–polymerase “click editors” that covalently attach diverse DNA cargos and install all 12 base substitutions plus >100-bp insertions or deletions in preclinical models [44]. By sidestepping HDR and expanding cargo size, these polymerase editors achieve higher precision with minimal indels, work in non-dividing cells, and simplify guide design relative to prime editing, making them a promising next-generation platform for versatile genome rewriting.

Recent breakthroughs also highlight the use of retron-based systems [45], CRISPR-associated transposases (CAST) [46], and mobile genetic elements [47] as novel gene editing platforms that may supplement or surpass CRISPR-Cas technologies in future applications.

Retroelement-based editors are non-LTR retrotransposons, exemplified by the silkworm R2 element, and are now being explored as CRISPR-retargetable tools for installing large DNA cargos. Like prime editors, they operate by target-primed reverse transcription (TPRT): a single-strand nick exposes a 3′-OH that serves as a primer for reverse-transcribing the retroelement RNA and copying it into the genome [48]. Recent cryo-EM snapshots of R2 have clarified how its endonuclease-RT complex recognizes DNA and initiates TPRT, and Cas9-nickase fusions have already redirected R2 integration to non-native sites, hinting at a modular “search-and-write” system for multi-kilobase inserts. Retroelements could thus complement CASTs by accommodating full gene payloads, yet their insertion precision, off-target profile, and cell-type-dependent efficiency remain to be rigorously benchmarked before therapeutic use [49].

TIGR-Tas represents a compact alternative to CRISPR discovered in bacteriophages and parasitic bacteria. This system utilizes guide RNAs with interspersed repeats (TIGR) and associated proteins (Tas) that recognize and bind DNA through a unique tandem spacer mechanism [50]. Unlike CRISPR, TIGR-Tas does not require PAM sequences and uses two spacers complementary to different DNA strands, ensuring high specificity. Tas proteins, such as TasR, contain nuclease domains and are structurally like IS110 transposases, making them promising for editing applications. Due to their compactness (on average 4-fold smaller than Cas9) and programmability, TIGR-Tas could become a powerful tool for therapeutic genome editing.

RNA editing harnesses programmable RNA-guided effectors — most prominently ADAR-recruiting guide RNAs and CRISPR type VI Cas13 or Cas7-11 nucleases — to install precise A→I (read as G), C→U or other single-nucleotide changes, modulate splicing, or selectively degrade transcripts, all without cutting DNA or creating permanent genomic scars. The toolbox now spans chemically modified AIMers and RESCUE/ REPAIR systems, catalytically re-engineered Cas13 deaminases, split-intein or mini-Cas platforms, and collateral-activity-free variants, delivering transcript-level specificity across a much broader target space than was achievable with early ADAR fusions alone [51]. Because edits are transient, dose-tunable, and independent of the cell cycle or homology-directed repair, RNA editing offers a safer, reversible route to correct pathogenic alleles, study gene function in post-mitotic tissues, and treat disorders where temporary, tissue-selective modulation is preferable to permanent genome alteration [52].

Epigenome editing repurposes nuclease-dead CRISPR platforms (most often dCas9, dCas12, or dCas13) as programmable DNA- or RNA-binding scaffolds to deliver writer, eraser, or reader domainsc — such as KRAB repressors, VP64/p300 activators, DNMT3A or TET1 methyl-modifiers, and histone acetyl-/deacetyl-transferases — enabling locus-specific, reversible control of chromatin marks and gene expression without altering the underlying DNA sequence. CRISPRa (activation) and CRISPRi (interference) systems, derived from catalytically inactivated Cas9 (dCas9), further expand this toolkit by enabling transcriptional activation or repression [53]. The CRISPRoff system induces heritable DNA methylation for long-term gene silencing [54], while dCas9-TET3CD counteracts pathological promoter hypermethylation via targeted hydroxymethylation [55]. Additionally, dCas9-HDAC and dCas9-HAT systems enable site-specific histone deacetylation or acetylation to modulate gene expression [56–58]. The development of light-inducible CRISPR systems such as paCas13 has provided temporal control over gene expression, enabling reversible RNA editing with pharmaceutical precision [59].

Recent advances in multiplexed transcriptional control have led to the optimization of Cas12a-based CRISPR activation (CRISPRa) systems. Unlike Cas9, Cas12a allows for facile multiplexing of guide RNAs from a single transcript, simplifying combinatorial perturbations. The development of nanobody-based recruitment systems using the ALFA tag and corresponding nanobody enables potent simultaneous activation of multiple endogenous genes. This multiplexing capability has been successfully demonstrated in genome-wide screens for identifying modulators of cellular growth and drug resistance, expanding the toolkit for functional genomics studies beyond traditional single-gene approaches [60].

The growing arsenal of epigenetic editors now includes split-intein mini-dCas fusions, scaffold-RNA assemblies, and CRISPR-Switchboard architectures, which combine multiple effectors to regulate complex gene networks. Innovations like SunTag for multiplexed regulation, base-free RNA editors, and CRISPRoff/ on systems for switchable DNA methylation enhance modularity, reduce off-target effects, and offer precise control over cellular states — such as development, reprogramming, and disease phenotypes — surpassing traditional genome or transcriptome editing in flexibility and specificity [52]. Together, these advancements highlight the transformative potential of epigenome editing in decoding regulatory circuits and engineering cell fates.

Mitochondrial genome-targeted tools now span two functional classes: programmable nucleases — mitoREs, mitoZFNs, mitoTALENs, mitoARCUS, and emerging Cas9-based designs — that cleave mutant genomes and exploit the organelle’s natural degradation pathways to “shift” heteroplasmy toward healthy copies. Complementing them are base-editing platforms such as DdCBE cytosine editors, TALED adenine editors, and the compact ZFDs, which install single-nucleotide conversions without double-strand breaks, enabling correction of both heteroplasmic and homoplasmic mutations and the creation of precise disease models [61]. Together, these systems offer a broad design palette that ranges from small, single-polypeptide meganucleases to split-enzyme TALE or zinc-finger fusions deliverable in AAV or LNP formats, allowing investigators to balance cargo size, targeting scope, and off-target risk for different tissues and species [62]. Their chief advantages are the ability to rescue bioenergetic function by selectively purging pathogenic mtDNA or by directly rewriting disease alleles, all while sidestepping nuclear editing and harnessing the high copy number of the mitochondrial genome to achieve therapeutically meaningful shifts with relatively modest editing efficiencies. Comprehensive genotoxicity assessments of programmable nucleases in human hematopoietic stem cells remain challenging due to limitations in current detection methods, with off-target activity varying depending on the nuclease platform, donor DNA, and targeted sequence, requiring case-by-case safety evaluations for clinical translation [63].

Advanced mitochondrial genome editing tools such as αDdCBEs have overcome the 5’-T constraint limitation, enabling targeting of over 150 previously inaccessible loci in the human mitochondrial genome with improved precision and reduced off-target effects [64].

## Gene therapy product delivery systems

The efficacy and safety of gene therapy largely depend on the delivery platform utilized, which determines the precision, stability, and tissue specificity of genetic material introduction. Given the diversity of therapeutic approaches — from delivering coding constructs and editing complexes to mRNA and small RNAs — the development of reliable and biocompatible vectors becomes a critical endeavor. Contemporary delivery systems encompass viral vectors, lipid nanoparticles, virus-like particles, exosomes, as well as hybrid and synthetic platforms. Each possesses unique advantages and limitations that define their application scope. The advancement of delivery technologies plays a pivotal role in the clinical translation of gene therapy, particularly when targeting hard-to-reach tissues and minimizing systemic adverse effects.

Viral vectors represent the most clinically validated platforms. Lentiviral vectors integrate transgenes into the host cell genome, which is particularly valuable for *ex vivo* modifications, though associated with potential insertional mutagenesis risk [65, 66].

Among recombinant AAV vectors, 12 serotypes provide tissue-specific tropism. All serotypes except AAV3B and AAV4 had high liver tropism [67]. AAV8 dominates blood disorder applications due to liver tropism [68], while novel engineered capsids like AAV. CAP-B10 demonstrate enhanced CNS targeting with reduced liver expression in both mice and primates [69]. AAV9 and AAVrh.10 cross the blood-brain barrier [70]. AAVhu.32-PLUS shows enhanced neurotropism [71]. AAV4 demonstrates pan-endothelial tropism while also targeting pancreatic beta cells [67]. Novel myotropic AAVs (MyoAAVs and AAVMYOs) are demonstrating enhanced muscle transduction efficiency [72]. Serotype 6 of adeno-associated virus (AAV6) exhibits high tropism for muscle tissue, making it particularly well-suited for gene delivery in muscle-targeted therapies [73]. AAV9, capable of crossing the blood-brain barrier, successfully delivers genetic material to neurons and glial cells, showing promising results for neurodegenerative diseases [74, 75]. Advanced AAV engineering includes the development of ancestral capsids like Anc80, which represents computationally predicted ancestors of contemporary AAV serotypes, offering improved transduction efficiency and broader tissue tropism [76].

Dual AAV vector systems have achieved efficient delivery of large therapeutic genes, with successful clinical results reported for OTOF gene delivery in mouse model with congenital hearing loss, demonstrating significant improvements in speech perception and partial hearing recovery [77].

Advanced viral vector production using chemically defined media and optimized feeding strategies has achieved clinical-scale manufacturing with improved vector titers and reduced contamination risks [78].

Lipid nanoparticles (LNPs) represent a new generation of nucleic acid delivery systems. Contemporary LNP formulations contain ionizable aminolipids, which facilitate endosomal escape [79]. Biodegradable LNPs with polyethylene glycol modifications demonstrate increased blood circulation time and reduced clearance by the mononuclear phagocyte system [80, 81]. pH-dependent ionizable lipids enable tissue-specific delivery, such as selective targeting, is also achieved through surface modification with ligands (GalNAc for hepatocytes, peptides for myocytes) and lipid composition optimization [82, 83]. Ionizable LNPs with degradable ionizable lipids have been shown to significantly reduce hepatic toxicity while improving endosomal escape [84].

Virus-like particles (VLPs) combine structural similarity to viral capsids while lacking infectious potential. The LVNP-CRISPR system, based on lentivirus-derived nanoparticles, directly delivers preassembled Cas9 protein and sgRNA (RNP complex) to target cells, ensuring transient expression of CRISPR/Cas9 components and potentially reducing off-target editing risks [85]. Third-generation PE-eVLPs enable efficient *in vivo* delivery of prime editor RNPs, achieving >45% editing in target cells with undetectable off-target activity and potential for low immunogenicity due to transient expression [86].

Exosomes, as natural mediates of intercellular communication, represent biocompatible vectors for nucleic acid delivery. Autologous exosomes derived from mesenchymal stem cells possess natural selective accumulation in inflammatory foci [87]. SKOV3 cancer cell-derived exosomes efficiently delivered CRISPR/Cas9 systems to ovarian tumor xenografts, demonstrating targeting dependent on their natural tropism to tumor tissues [88].

SEND (selective endogenous encapsidation for cellular delivery) represents an innovative delivery platform based on modification of PEG10 protein — a natural mammalian protein that is an evolutionary analog of retroviral structural Gag protein. PEG10 selectively packages and secretes RNA with specific structural motifs through a mechanism analogous to retroviral encapsidation [89]. The SEND system demonstrates promising histocompatibility and limited immunogenicity, potentially addressing key biocompatibility limitations of existing platforms.

Recent advances in LNP technology include SORT (selective organ targeting) systems achieving ~65% editing efficiency in lung endothelial cells and ~40% in lung epithelial cells with sustained protein expression for over 7 days following intravenous administration [90]. Hybrid delivery systems combine advantages of different platforms. LNP-AAV hybrids encapsulate AAV in liposomes, protecting against neutralizing antibodies and enabling repeat dosing. Exosome-enriched AAV (exo-AAV) shows enhanced transduction efficiency and reduced immunogenicity compared to native AAV [91, 92]. Polymeric nanoparticles based on biodegradable polymers (PLGA, PEI) present an alternative to LNPs with controlled release profiles and expanded surface modification capabilities [93]. Polymersomes containing pH-sensitive block copolymers enable efficient nucleic acid delivery to central nervous system cells [94].

Ultrasound-mediated blood-brain barrier modulation with microbubbles represents a noninvasive method for delivering gene therapeutic agents to the CNS. Focused ultrasound with microbubbles temporarily increases BBB permeability, which in combination with intravenous vector administration significantly enhances transduction efficiency of neurons and glial cells [95, 96]. Additionally, strategies like magnetofection [97], are under active exploration to enhance delivery precision across the blood-brain barrier and immune-privileged organs. Magnetofection combined with focused ultrasound has emerged as a promising strategy for non-invasive blood-brain barrier modulation, enabling enhanced gene therapy delivery to CNS tissues while minimizing systemic exposure.

Nucleic acid drugs like siRNA or antisense oligonucleotides can be also attached to single-stranded extensions on the DNA nanostructure [98, 99].

CAR-T cell therapy is expanding beyond oncology. Breakthrough work using CAR constructs targeting fibroblast activation protein showed significant reduction in myocardial fibrosis and improved cardiac function in heart failure models [100]. Transient antifibrotic CAR constructs encoded by modified mRNA and delivered via CD5-targeted LNPs induced CAR-T cell formation *in vivo*, ameliorating fibrosis and cardiac function deterioration in a murine model with increased cardiac afterload [101].

The Figure presents an overview of gene therapy and genome editing strategies.

**Figure:**
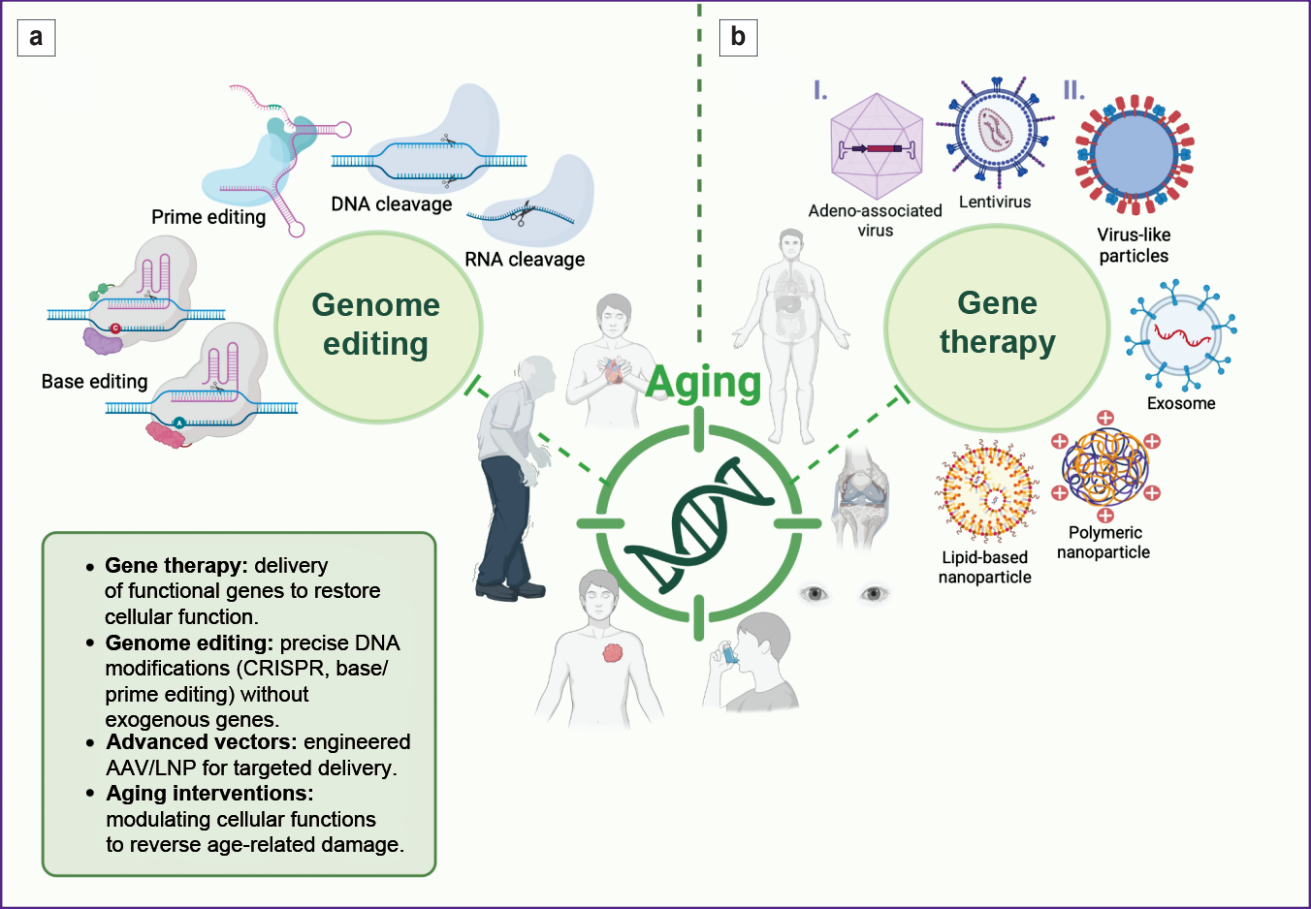
Gene therapy and genome editing strategies: (a) core approaches in genome editing. Gene therapy involves the delivery of functional genes or regulatory elements to restore or enhance cellular functions, whereas genome editing enables precise modifications of endogenous DNA sequences without the insertion of exogenous genes. Key advancements include: CRISPR/Cas systems; base editing; prime editing; RNA editing; (b) delivery systems: enabling precision and safety. Efficient delivery remains critical for therapeutic efficacy. Current platforms include: ***I. Viral vectors:** adeno-associated virus* (AAV): tissue-specific serotypes and engineered variants; *lentiviruses*: integrate therapeutic genes into the host genome, making them ideal for *ex vivo* applications but carrying risks of insertional mutagenesis. ***II. Non-viral systems:** lipid nanoparticle* (LNP): deliver mRNA or CRISPR ribonucleoproteins; pH-sensitive formulations enhance endosomal escape and allow organ-specific targeting (e.g., GalNAc-LNPs for hepatocytes); *exosomes*: naturally derived vesicles with low immunogenicity, optimized for targeted delivery in tumors or inflamed tissues; *polymeric nanoparticle and SEND systems*: virus-like particles and PEG10-based SEND platforms transiently express genome editors, thereby reducing off-target and long-term expression risks

## Conclusions

The comprehensive analysis of contemporary gene therapy technologies reveals a field characterized by significant technical achievements alongside persistent translational challenges. Gene replacement strategies using therapeutic mRNA, siRNA-mediated silencing approaches, and traditional gene delivery methods have established foundational principles for nucleic acid therapeutics, while the subsequent development of precision genome editing platforms has expanded the therapeutic toolkit. The progression from basic gene delivery to sophisticated editing systems, including base editors, prime editing platforms, and DNA polymerase-based tools, demonstrates incremental advances in achieving targeted genetic modifications with improved specificity profiles.

Delivery system optimization, particularly through engineered AAV serotypes and ionizable lipid nanoparticles, has addressed some critical barriers to therapeutic efficacy, yet substantial limitations persist.

Manufacturing scalability remains a significant constraint, with complex production requirements for viral vectors and ribonucleoprotein assemblies creating economic barriers to widespread implementation. Current cell and gene therapy manufacturing faces a critical capacity utilization challenge, with many CDMOs (contract development and manufacturing organizations) operating at less than 50% capacity while demand remains concentrated in clinical trials rather than commercial production [102]. Quality by design approaches combined with ultra scale-down technologies are accelerating AAV bioprocess development, addressing the time-intensive nature of current manufacturing scale-up procedures [103].

Safety considerations continue to present formidable challenges, particularly regarding long-term consequences of permanent genomic modifications, potential off-target effects in complex genomic contexts, and immunological responses to vector components or editing machinery. The integration of physics-informed AI models such as Elektrum has improved CRISPR safety prediction by accurately forecasting off-target effects through biophysical modeling of Cas9 kinetics, achieving superior performance compared to existing machine learning approaches [104].

Regulatory agencies have established accelerated approval pathways for gene therapies.

The delivery of therapeutic agents across physiological barriers, especially to privileged tissues such as the central nervous system, remains technically demanding despite advances in vector engineering and permeabilization strategies. Post-mitotic tissue targeting presents additional complications, with limited regenerative capacity constraining therapeutic windows and editing efficiency. Resistance mechanisms, dosing optimization, and the development of standardized protocols for patient selection require systematic investigation before these approaches can achieve routine clinical application.

The integration of computational approaches for predictive analysis offers potential solutions to some technical limitations, yet empirical validation remains essential for establishing therapeutic reliability.

Current evidence suggests that while gene therapy technologies have demonstrated proof-of-concept in selected applications, their broader clinical implementation faces substantial technical, regulatory, and economic hurdles that require sustained research investment and careful evaluation of risk-benefit profiles across diverse therapeutic contexts.
